# HOW I DO IT: Cushing’s disease—selective adenomectomy via an endoscopic transsphenoidal approach

**DOI:** 10.1007/s00701-024-06078-y

**Published:** 2024-06-06

**Authors:** N. Phillips, P. Nix, M. De Santos

**Affiliations:** Leeds General Infirmary, Leeds, UK

**Keywords:** Cushing's Disease, Selective adenomectomy, Endoscopic, Trans-sphenoidal, technique, Nuances

## Abstract

**Background:**

An ACTH-secreting pituitary adenoma is the most common cause of excessive endogenous glucocorticoid production resulting in Cushing’s Syndrome. A multidisciplinary approach is paramount. Selective adenomectomy is the treatment of choice.

**Method:**

Endoscopic transnasal transsphenoidal approach to the tumour, along with techniques for resection, are demonstrated.

**Conclusion:**

Endoscopic transsphenoidal approaches with its magnified view of the pituitary gland allows precise microsurgical dissection during selective adenomectomy. This technique increases the possibility of proving a gross total resection, leading to clinical and biochemical cure in these patients.

**Supplementary Information:**

The online version contains supplementary material available at 10.1007/s00701-024-06078-y.

## Relevant surgical anatomy

Understanding the standard endonasal rhinological anatomy is paramount.

A conchal sphenoid sinus necessaries staying in the midline with reliance on neuronavigation to identify the ICAs bilaterally and defining the limits of the sella. The typical surface anatomy—optic nerves, and MCR couldn’t be identified.

**Fig. 1:**
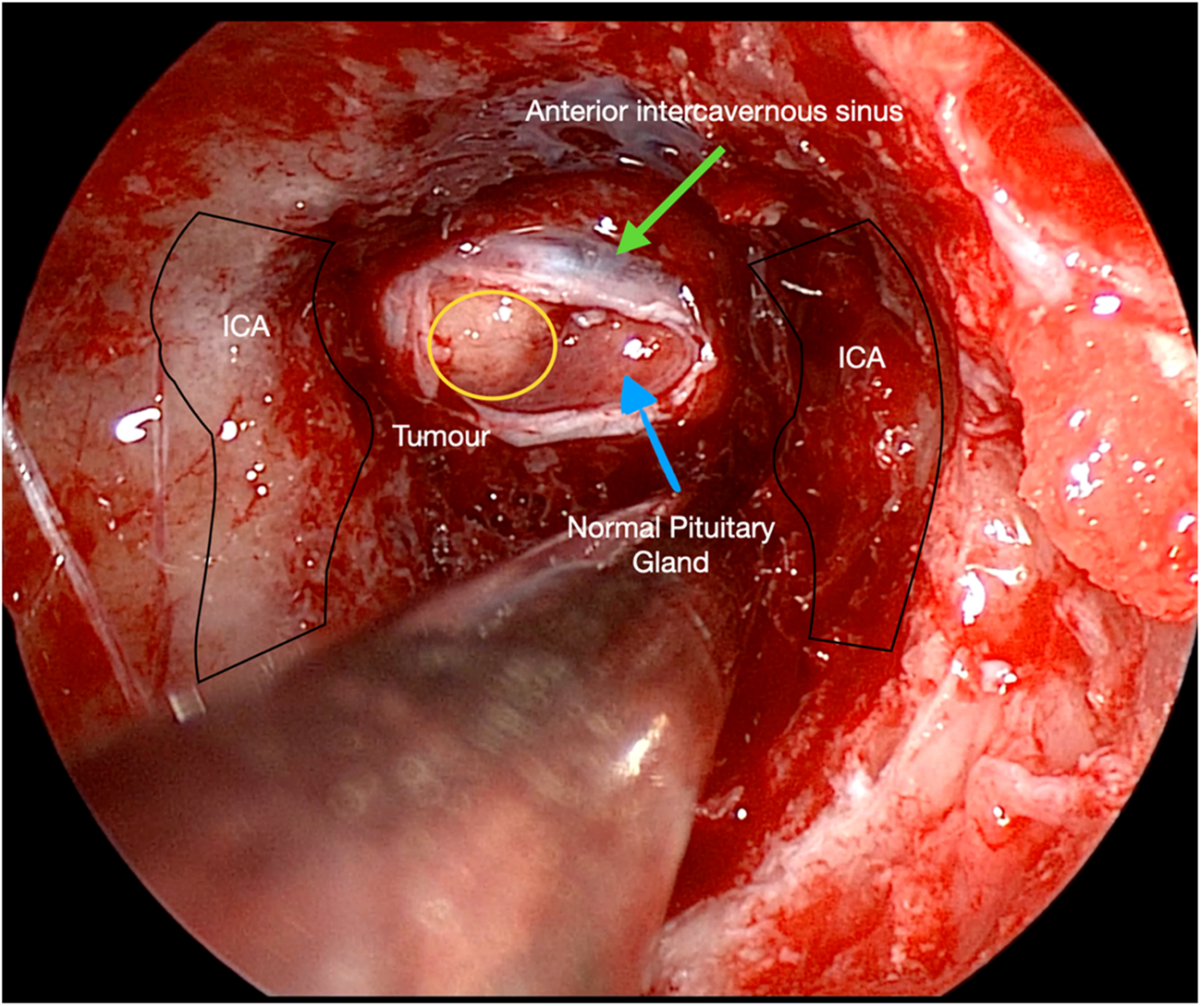
An endoscopic view showing essential intra-sphenoidal anatomy. Internal Carotid arteries (ICA), a right-sided pituitary microadenoma (yellow)

**Fig. 2:**
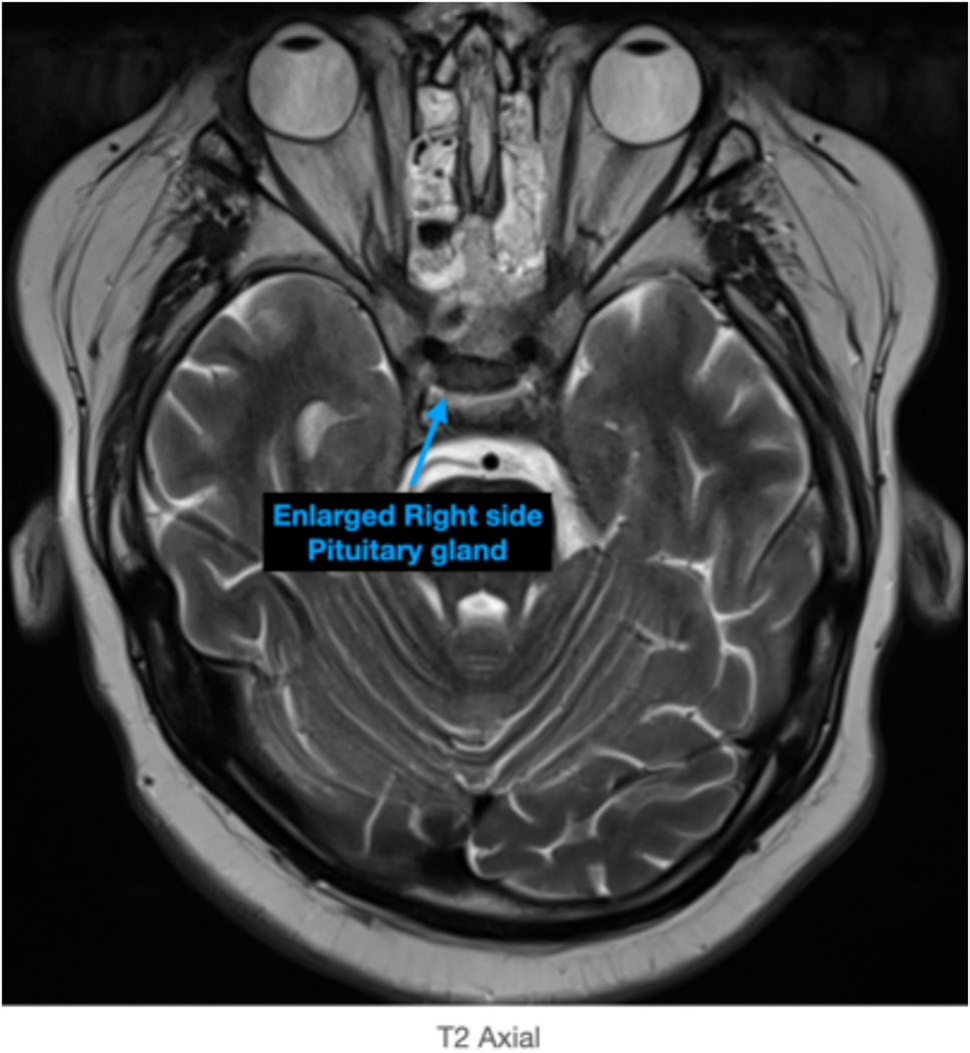
Axial MRI T2 demonstrates a right-sided pituitary gland enlargement correlating to the pituitary microadenoma

**Fig. 3:**
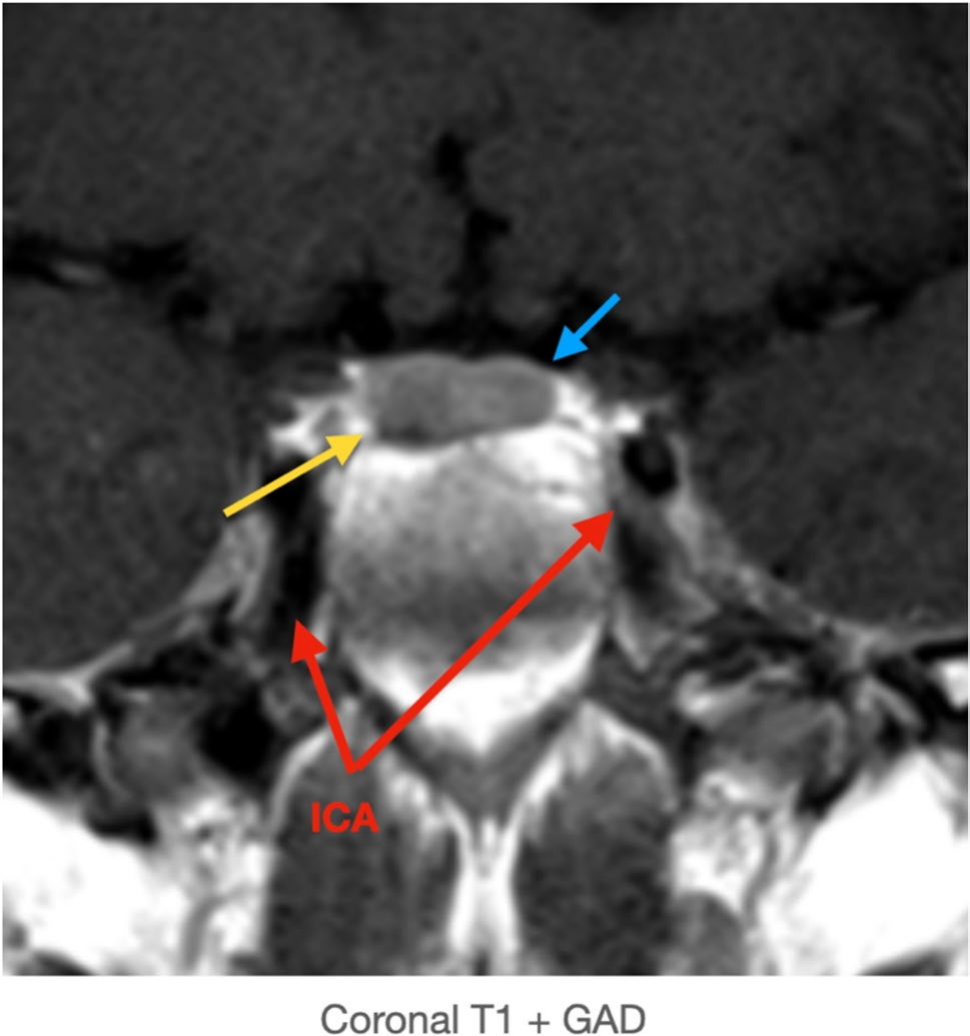
Coronal T1 + GAD demonstrating the disproportionately enlarged right pituitary gland - microadenoma (yellow arrow), normal pituitary gland (blue arrow), and the internal carotid arteries (red arrows)

**Fig. 4:**
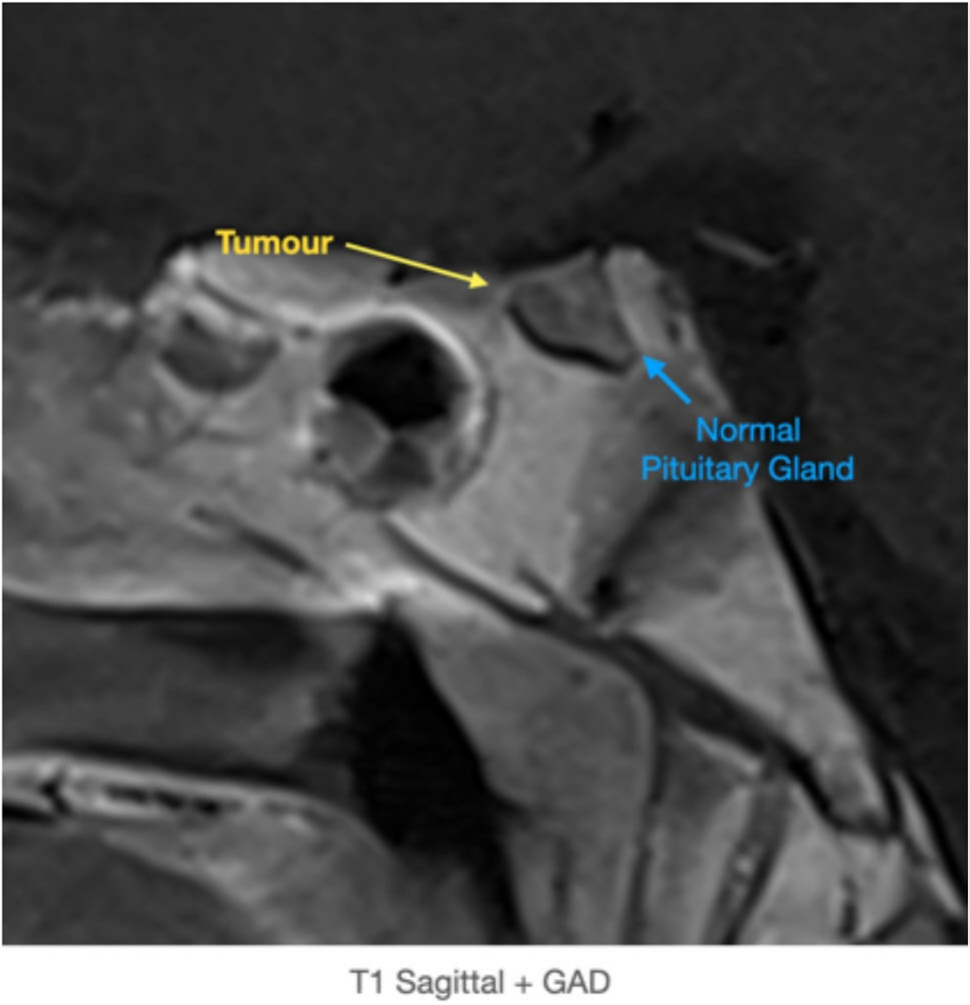
T1 Sagittal + GAD demonstrating the pituitary microadenoma (yellow arrow) and normal pituitary gland (blue arrow). The conchal sphenoidal sinus can be appreciated

Major landmarks are should in Figs. 1, 2, 3 and 4.

## Description of the technique

### Scans & navigation

Preoperative Tri-planar MRI scan with Pituitary protocol -T2, CISS T1 pre and post sequences. Tumours challenging to visualise undergo dynamic MRI imaging. Fine-cut CT of the paranasal sinuses is acquired to understand the nasal and sphenoidal anatomy and variations. Electromagnetic neuronavigation (Medtronic) is used.

### Anaesthesia

Total Intravenous anaesthesia (TIVA), pre-and post-operative hydrocortisone, tranexamic acid (TXA) and meticulous BP control with IV Labetalol were used to achieve stable anaesthesia and adequate haemostasis. TXA and Labetalol aren’t used for standard pituitaries. Cushing’s related hypertension can be intractable and intraoperative IV Labetalol is quite helpful. The insertion of cophenylcaine nasal spray is used as a vasoconstrictor and aid haemostasis. Coughless extubation is essential to reduce post-operative haemorrhage since the nasal cavity isn’t packed after the procedure [5].

### Positioning

Supine with the head elevated to 15°. Meticulous padding of pressure points is essential as Cushingoid patients bruise easily.

### Technique

Adrenaline-soaked patties (1:000) intranasally aid haemostasis prior to neuronavigation registration.

The intranasal anatomy is defined using a zero-degree endoscope.

We routinely resect the right middle turbinate to increase the working channel with care taken not to disrupt the mucosa posteriorly containing the nasoseptal branch of the sphenopalatine artery. The superior turbinate is lateralized, and the choana and the sphenoid ostium are identified [5].

A Hadad- Bassagasteguy flap is performed in all cases by our ENT colleagues, avoiding injury to the olfactory epithelium [2, 5].

A posterior septectomy is performed with a microdebrider, with the removal of all exposed cartilage. This decreases the exposed tissue required for healing, which is a challenge in Cushing’s patients. Additionally, it facilitates ease of instrument placement along the operative corridor.

A two-surgeon technique is employed. One surgeon drives the scope and irrigates in the left nostril while the primary surgeon gains access to the sella turcica and performs the hypophysectomy.

In this case, the sella was non-pneumatized, which required extensive drilling. EM neuronavigation is used. A Medtronic endonasal drill enabled drilling of the sphenoid bone, facilitating identification of the sella. Fatty infiltration of the sphenoid bone facilitated relatively easy drilling. Kerrison rongeurs were used to remove the egg-shelled bone and complete the sellar exposure.

After bony exposure, a right-sided pituitary enlargement was seen and a H-shaped durotomy performed. A plane between the dura and the pituitary gland is developed. The anterior intercavernous sinus was identified.

A sharp incision medial to the tumour in the plane of the pseudocapsule is performed.

The plane of the tumour’s pseudocapsule was meticulously developed. Tumour removal was done enbloc. The medial wall of the right cavernous sinus is visualised and remaining tumour removed.

No further lesion was noted after further exploration of the gland.

Post resection, haemostasis is achieved warm irrigation and Floseal (Baxter). Inlay spongistan graft placed and secured with fibrin glue. The nasoseptal flap was positioned onto the sphenoid floor and sella with direct contact with all bony elements. Nasopore forte (Polyganics)was used to buttress the flap [4, 5].

Coughless extubation was performed, with the patient nursed upright at approximately 60 degrees [5].

## Indications

The presented case illustrated our standard surgical approach to managing clinically functional discrete tumours.

In particular, the beforementioned selective adenomectomy is an ideal procedure for these well-circumscribed secretory tumours identified on MRI [3].

This resection method provided little disruption to the normal pituitary gland while obtaining a gross total resection and ensuring a high likelihood of obtaining a biochemical cure.

Selective hypophysectomy done meticulously is the gold standard treatment for secretory pituitary adenomas [3].

## Limitations

A selective adenomectomy cannot be planned in cases where there is no radiological demonstration of the tumour.

## How to avoid complications

CT of the paranasal sinuses is essential to identify any abnormal nasal anatomy, especially relating to the sphenoid sinus, aeration patterns and, optic nerves. Sphenoid sinus septae that attach to the carotid canal should be noted [3, 4].

Identification of the distance between the intracavernous carotid arteries is best appreciated on MRI.

Intraoperative doppler is helpful in cases where dehiscence is present or if neuronavigation fails.

The most common complication in these endonasal cases is CSF leaks. Our unit leak rate for non-extended approaches is less than 1%, which is avoided by the routine use of the nasoseptal flap [4].

Patients with non-pneumatised sphenoid sinuses pose a particular challenge endonasally because it has to relies heavily on neuronavigation. Where neuronavigation fails, staying in the midline along the keel of the sphenoid will invariably lead the sella.

Gentle manipulation of the pituitary gland is paramount to minimise postoperative pituitary dysfunction.

Bleeding can be troublesome because of the fragile tissues in Cushings. It can also arise from the anterior intercavernous sinus. Venous bleeding is best controlled by elevating the head of the bed, compression with patties, warm irrigation, and the use of Floseal(Baxter) [4]. The Aquamantys (Medtronic) bipolar may be used for more stubborn bleeding.

## Specific perioperative considerations

A multidisciplinary approach is critical which included a throughout endocrinological and radiological evaluation of pituitary function along with evaluating ectopic sources of the ACTH—Cushing Syndrome. Inferior petrosal sinus sampling is performed in cases of MRI negative Cushing’s disease. In these cases, a partial hypophysectomy directed by IPSS is a surgical option and should be discussed [6].

Formal Goldman Visual Field assessments (GVF) perioperatively is required in cases with visual disturbance.

Postoperatively, hydrocortisone replacement is done while observing for signs of pituitary dysfunction. Day 1, 6 a.m., cortisol is used to define the response to surgery. Routine GVF and 6-month scan postoperative MRI are performed to assess for residual tumour [1].

Patient are education about the loss of smell and taste (temporarily), CSF leaks, and a regime for steroid supplementation during sick days. Rhinological review is at 6 weeks [5].

Patients are typically discharged by day two.

## Supplementary Information

Below is the link to the electronic supplementary material.

Supplementary file1 (MP4 390149 KB)

## Data Availability

All data underlying this paper is available as part of this article and no additional sources of data are required.

## References

[1] Fleseriu M, Auchus R, Bancos I, Ben-Shlomo A, Bertherat J, Biermasz NR et al (2021) Consensus on diagnosis and management of Cushing’s disease: a guideline update. Lancet Diabetes Endocrinol 9:847–875 34687601 10.1016/S2213-8587(21)00235-7 PMC8743006

[2] Hadad G, Bassagasteguy L, Carrau RL, Mataza JC, Kassam A, Snyderman CH et al (2006) A novel reconstructive technique after endoscopic expanded endonasal approaches: vascular pedicle nasoseptal flap. Laryngoscope [Internet] 116:1882–6. Available from: https://pubmed.ncbi.nlm.nih.gov/17003708/. Accessed 5 Oct 2023 10.1097/01.mlg.0000234933.37779.e4 17003708

[3] Lonser RR, Nieman L, Oldfield EH (2017) Cushing’s disease: pathobiology, diagnosis, and management. J Neurosurg [Internet] 126:404–17. Available from: https://pubmed.ncbi.nlm.nih.gov/27104844/. Accessed 5 Oct 2023 10.3171/2016.1.JNS152119 27104844

[4] Nix P, Tyagi A, Phillips N (2016) Evolution of a UK endoscopic anterior skull base pituitary service - the first one hundred and twenty-three patients: Our Experience. Clin Otolaryngol [Internet] 41:289–93. Available from: https://pubmed.ncbi.nlm.nih.gov/26243005/. Accessed 5 Oct 2023 10.1111/coa.12514 26243005

[5] Phillips N, Nix P (2016) How I do it - endoscopic endonasal approach for pituitary tumour. Acta Neurochir (Wien) [Internet] 158:1983–5. 10.1007/s00701-016-2916-z 27526186 10.1007/s00701-016-2916-z PMC5025490

[6] Sabahi M, Shahbazi T, Maroufi SF, Vidal K, Recinos PF, Kshettry VR et al (2022) MRI–negative cushing’s disease: A review on therapeutic management. World Neurosurg [Internet] 162:126-137.e1. 10.1016/j.wneu.2022.03.076 35338018 10.1016/j.wneu.2022.03.076

